# Variation in monitoring and treatment policies for intracranial hypertension in traumatic brain injury: a survey in 66 neurotrauma centers participating in the CENTER-TBI study

**DOI:** 10.1186/s13054-017-1816-9

**Published:** 2017-09-06

**Authors:** Maryse C. Cnossen, Jilske A. Huijben, Mathieu van der Jagt, Victor Volovici, Thomas van Essen, Suzanne Polinder, David Nelson, Ari Ercole, Nino Stocchetti, Giuseppe Citerio, Wilco C. Peul, Andrew I. R. Maas, David Menon, Ewout W. Steyerberg, Hester F. Lingsma, on behalf of the CENTER-TBI investigators, Hadie Adams, Masala Alessandro, Judith Allanson, Krisztina Amrein, Norberto Andaluz, Nada Andelic, Nanni Andrea, Lasse Andreassen, Audny Anke, Anna Antoni, Hilko Ardon, Gérard Audibert, Kaspars Auslands, Philippe Azouvi, Camelia Baciu, Andrew Bacon, Rafael Badenes, Trevor Baglin, Ronald Bartels, Pál Barzó, Ursula Bauerfeind, Ronny Beer, Francisco Javier Belda, Bo-Michael Bellander, Antonio Belli, Rémy Bellier, Habib Benali, Thierry Benard, Maurizio Berardino, Luigi Beretta, Christopher Beynon, Federico Bilotta, Harald Binder, Erta Biqiri, Morten Blaabjerg, Lund Stine Borgen, Pierre Bouzat, Peter Bragge, Alexandra Brazinova, Felix Brehar, Camilla Brorsson, Andras Buki, Monika Bullinger, Veronika Bučková, Emiliana Calappi, Peter Cameron, Guillermo Carbayo Lozano, Elsa Carise, K. Carpenter, Ana M. Castaño-León, Francesco Causin, Giorgio Chevallard, Arturo Chieregato, Giuseppe Citerio, Maryse Cnossen, Mark Coburn, Jonathan Coles, Jamie D. Cooper, Marta Correia, Amra Covic, Nicola Curry, Endre Czeiter, Marek Czosnyka, Claire Dahyot-Fizelier, François Damas, Pierre Damas, Helen Dawes, Véronique De Keyser, Francesco Della Corte, Bart Depreitere, Shenghao Ding, Diederik Dippel, Kemal Dizdarevic, Guy-Loup Dulière, Adelaida Dzeko, George Eapen, Heiko Engemann, Ari Ercole, Patrick Esser, Erzsébet Ezer, Martin Fabricius, Valery L. Feigin, Junfeng Feng, Kelly Foks, Francesca Fossi, Gilles Francony, Janek Frantzén, Ulderico Freo, Shirin Frisvold, Alex Furmanov, Pablo Gagliardo, Damien Galanaud, Guoyi Gao, Karin Geleijns, Alexandre Ghuysen, Benoit Giraud, Ben Glocker, Pedro A. Gomez, Francesca Grossi, Russell L. Gruen, Deepak Gupta, Juanita A. Haagsma, Ermin Hadzic, Iain Haitsma, Jed A. Hartings, Raimund Helbok, Eirik Helseth, Daniel Hertle, Sean Hill, Astrid Hoedemaekers, Stefan Hoefer, Peter J. Hutchinson, Asta Kristine Håberg, Bram Jacobs, Ivan Janciak, Koen Janssens, Ji-yao Jiang, Kelly Jones, Jean-Pierre Kalala, Konstantinos Kamnitsas, Mladen Karan, Jana Karau, Ari Katila, Maija Kaukonen, David Keeling, Thomas Kerforne, Naomi Ketharanathan, Johannes Kettunen, Riku Kivisaari, Angelos G. Kolias, Bálint Kolumbán, Erwin Kompanje, Daniel Kondziella, Lars-Owe Koskinen, Noémi Kovács, Ferenc Kálovits, Alfonso Lagares, Linda Lanyon, Steven Laureys, Martin Lauritzen, Fiona Lecky, Christian Ledig, Rolf Lefering, Valerie Legrand, Jin Lei, Leon Levi, Roger Lightfoot, Hester Lingsma, Dirk Loeckx, Angels Lozano, Roger Luddington, Chantal Luijten-Arts, I. R. Maas Andrew, Stephen MacDonald, Charles MacFayden, Marc Maegele, Marek Majdan, Sebastian Major, Alex Manara, Pauline Manhes, Geoffrey Manley, Didier Martin, Costanza Martino, Armando Maruenda, Hugues Maréchal, Dagmara Mastelova, Julia Mattern, Catherine McMahon, Béla Melegh, David Menon, Tomas Menovsky, Cristina Morganti-Kossmann, Davide Mulazzi, Manuel Mutschler, Holger Mühlan, Ancuta Negru, David Nelson, Eddy Neugebauer, Virginia Newcombe, Quentin Noirhomme, József Nyirádi, Mauro Oddo, Annemarie Oldenbeuving, Matej Oresic, Fabrizio Ortolano, Aarno Palotie, Paul M. Parizel, Adriana Patruno, Jean-François Payen, Natascha Perera, Vincent Perlbarg, Paolo Persona, Wilco Peul, Nicolas Pichon, Henning Piilgaard, Anna Piippo, Sébastien Pili Floury, Matti Pirinen, Horia Ples, Suzanne Polinder, Inigo Pomposo, Marek Psota, Pim Pullens, Louis Puybasset, Arminas Ragauskas, Rahul Raj, Malinka Rambadagalla, Veronika Rehorčíková, Jonathan Rhodes, Sylvia Richardson, Samuli Ripatti, Saulius Rocka, Nicolas Rodier, Cecilie Roe, Olav Roise, Gerwin Roks, Pauline Romegoux, Jonathan Rosand, Jeffrey Rosenfeld, Christina Rosenlund, Guy Rosenthal, Rolf Rossaint, Sandra Rossi, Tim Rostalski, Daniel Rueckert, Felix Ruiz de Arcaute, Martin Rusnák, Marco Sacchi, Barbara Sahakian, Juan Sahuquillo, Oliver Sakowitz, Francesca Sala, Paola Sanchez-Pena, Renan Sanchez-Porras, Janos Sandor, Edgar Santos, Nadine Sasse, Luminita Sasu, Davide Savo, Inger Schipper, Barbara Schlößer, Silke Schmidt, Annette Schneider, Herbert Schoechl, Guus Schoonman, Rico Frederik Schou, Elisabeth Schwendenwein, Michael Schöll, Özcan Sir, Toril Skandsen, Lidwien Smakman, Dirk Smeets, Peter Smielewski, Abayomi Sorinola, Emmanuel Stamatakis, Simon Stanworth, Katrin Stegemann, Nicole Steinbüchel, Robert Stevens, William Stewart, Ewout W. Steyerberg, Nino Stocchetti, Nina Sundström, Anneliese Synnot, József Szabó, Jeannette Söderberg, Fabio Silvio Taccone, Viktória Tamás, Päivi Tanskanen, Alexandru Tascu, Mark Steven Taylor, Braden Te Ao, Olli Tenovuo, Guido Teodorani, Alice Theadom, Matt Thomas, Dick Tibboel, Christos Tolias, Jean-Flory Luaba Tshibanda, Cristina Maria Tudora, Peter Vajkoczy, Egils Valeinis, Wim Van Hecke, Dominique Van Praag, Dirk Van Roost, Eline Van Vlierberghe, Thijs Vande Vyvere, Audrey Vanhaudenhuyse, Alessia Vargiolu, Emmanuel Vega, Jan Verheyden, Paul M. Vespa, Anne Vik, Rimantas Vilcinis, Giacinta Vizzino, Carmen Vleggeert-Lankamp, Victor Volovici, Peter Vulekovic, Zoltán Vámos, Derick Wade, Kevin K. W. Wang, Lei Wang, Eno Wildschut, Guy Williams, Lisette Willumsen, Adam Wilson, Lindsay Wilson, Maren K. L. Winkler, Peter Ylén, Alexander Younsi, Menashe Zaaroor, Zhiqun Zhang, Zelong Zheng, Fabrizio Zumbo, Stefanie de Lange, Godard C. W. de Ruiter, Hugo den Boogert, Jeroen van Dijck, Thomas A. van Essen, Caroline van Heugten, Mathieu van der Jagt, Joukje van der Naalt

**Affiliations:** 1 Center for Medical Decision Making, Department of Public Health, Erasmus MC, Rotterdam, The Netherlands; 2 Department of Intensive Care, Erasmus MC, Rotterdam, The Netherlands; 3 Department of Neurosurgery, Erasmus MC, Rotterdam, The Netherlands; 4 Department of Neurosurgery, Leiden University Medical Center, Leiden, The Netherlands; 5 Department of Physiology and Pharmacology, Section of Perioperative Medicine and Intensive Care, Karolinska Institutet, Stockholm, Sweden; 6 Division of Anesthesia, University of Cambridge, Addenbrooke’s Hospital, Cambridge, UK; 7 Department of Pathophysiology and Transplants, University of Milan, Milan, Italy; 8 Fondazione IRCCS Ca’ Granda – Ospedale Maggiore Policlinico, Department of Anesthesia and Critical Care, Neuroscience Intensive Care Unit, Milan, Italy; 9 School of Medicine and Surgery, University of Milano Bicocca, Milan, Italy; 10 Neurointensive Care Unit, San Gerardo Hospital, ASST-Monza, Monza, Italy; 11 Department of Neurosurgery, Haaglanden Medical Center, The Hague, The Netherlands; 12 Department of Neurosurgery, Antwerp University Hospital and University of Antwerp, Edegem, Belgium; 13 Department of Medical Statistics and Bioinformatics, Leiden University Medical Center, Leiden, The Netherlands

**Keywords:** Traumatic brain injury, Intracranial hypertension, ICP, ICU, Comparative effectiveness research, Survey

## Abstract

**Background:**

No definitive evidence exists on how intracranial hypertension should be treated in patients with traumatic brain injury (TBI). It is therefore likely that centers and practitioners individually balance potential benefits and risks of different intracranial pressure (ICP) management strategies, resulting in practice variation. The aim of this study was to examine variation in monitoring and treatment policies for intracranial hypertension in patients with TBI.

**Methods:**

A 29-item survey on ICP monitoring and treatment was developed on the basis of literature and expert opinion, and it was pilot-tested in 16 centers. The questionnaire was sent to 68 neurotrauma centers participating in the Collaborative European Neurotrauma Effectiveness Research in Traumatic Brain Injury (CENTER-TBI) study.

**Results:**

The survey was completed by 66 centers (97% response rate). Centers were mainly academic hospitals (*n* = 60, 91%) and designated level I trauma centers (*n* = 44, 67%). The Brain Trauma Foundation guidelines were used in 49 (74%) centers. Approximately 90% of the participants (*n* = 58) indicated placing an ICP monitor in patients with severe TBI and computed tomographic abnormalities. There was no consensus on other indications or on peri-insertion precautions. We found wide variation in the use of first- and second-tier treatments for elevated ICP. Approximately half of the centers were classified as using a relatively aggressive approach to ICP monitoring and treatment (*n* = 32, 48%), whereas the others were considered more conservative (*n* = 34, 52%).

**Conclusions:**

Substantial variation was found regarding monitoring and treatment policies in patients with TBI and intracranial hypertension. The results of this survey indicate a lack of consensus between European neurotrauma centers and provide an opportunity and necessity for comparative effectiveness research.

**Electronic supplementary material:**

The online version of this article (doi:10.1186/s13054-017-1816-9) contains supplementary material, which is available to authorized users.

## Background

Secondary brain injury associated with elevated intracranial pressure (ICP) is an important cause of mortality and morbidity in patients with severe traumatic brain injury (TBI) [1]. Therefore, identifying high ICP and optimizing its management is believed to be critically important. Yet, no definitive evidence exists on how ICP should be monitored and treated [2]. Patient and treatment heterogeneity make conducting randomized controlled trials (RCTs) challenging. On one hand, the majority of RCTs done to date have produced nonsignificant findings [3]. On the other hand, observational studies, which are easier to conduct, are at risk for confounding by indication, hampering causal inference [4, 5].

In the absence of conclusive evidence, treatment policy is usually based on local practices, individual preferences, and resource availability [6–9]. It is likely that centers and practitioners individually balance potential benefits and risks of different ICP management strategies, which may result in some centers being relatively aggressive and others being more conservative in their treatment policies.

A novel and promising approach in estimating treatment effectiveness is to exploit the existing variation by comparing standard practices between different centers or countries, which is referred to as comparative effectiveness research (CER) [10, 11]. The Collaborative European Neurotrauma Effectiveness Research in Traumatic Brain Injury (CENTER-TBI) study (grant 602150) is currently recruiting and will use CER methodology to study treatment effectiveness of ICP management [10]. As a first step, we examined self-perceived practices of ICP monitoring and associated treatment policies by sending a survey to the centers participating in the CENTER-TBI study. Because previous European survey studies that addressed ICP management were published more than 10 years ago [12, 13], this study will provide an up-to-date overview of ICP management in Europe. Topics identified as showing substantial between-center variation that are plausibly associated with patient outcome will be selected for CER, and their treatment effectiveness can be studied once the CENTER-TBI patient-level data become available.

## Methods

### Study sample

All centers participating in the prospective, longitudinal, observational CENTER-TBI study (https://www.center-tbi.eu/) were asked to complete a set of questionnaires on structures and processes of care for patients with TBI. Questionnaires were sent to 71 centers in 20 countries between 2014 and 2015 [14]. Three centers dropped out of the CENTER-TBI study, resulting in 68 eligible centers from Austria (*n* = 2), Belgium (*n* = 4), Bosnia and Herzegovina (*n* = 2), Denmark (*n* = 2), Finland (*n* = 2), France (*n* = 7), Germany (*n* = 4), Hungary (*n* = 2), Israel (*n* = 2), Italy (*n* = 9), Lithuania (*n* = 2), Latvia (*n* = 3), The Netherlands (*n* = 7), Norway (*n* = 2), Romania (*n* = 1), Serbia (*n* = 1), Spain (*n* = 4), Sweden (*n* = 2), the United Kingdom (*n* = 9), and Switzerland (*n* = 1).

### Questionnaire development and administration

A set of questionnaires designed to measure structure and process of TBI care was developed on the basis of available literature and expert opinion. These questionnaires were comprehensively described in a previous publication [14]. Pilot testing was undertaken in 16 of the participating centers, and feedback was incorporated into the final questionnaire design.

One of the questionnaires contained 29 questions on ICP monitoring and treatment at the intensive care unit (ICU) (Additional file 1). In most questions, we explicitly asked for the “general policy,” which was defined as the treatment or monitoring modality estimated to be used in more than 75% of patients, recognizing that there might be exceptions. In some questions, we asked for quantitative estimations. The representatives of the centers could indicate how often they used a particular monitoring or treatment strategy (never = 0–10%, rarely = 10–30%, sometimes = 30–70%, frequently = 70–90%, always = 90–100%). The options “frequently” and “always” were interpreted as representing the general policy, in line with a previous report [15]. All definitions used in the questionnaire are described in Additional file 2.

### Analyses

We calculated frequencies and percentages for all variables related to the number of responders for that variable. We examined factors associated with a relatively aggressive ICP monitoring and treatment strategy with the chi-square test or Fisher’s exact test as appropriate. Centers were classified as being relatively aggressive if they (a) place an ICP monitor in patients with a Glasgow Coma Scale (GCS) score ≤ 8 and an abnormal head computed tomographic (CT) scan, and (b) if they generally perform at least one of three second-tier treatments that represent a maximum therapy intensity (barbiturates, decompressive craniectomy, and hypothermia < 35 °C) [16].

We examined whether there were differences between and within geographic regions in the use of first- and second-tier treatments. Countries were divided into seven geographic regions (Northern Europe, Western Europe, United Kingdom, Southern Europe, Eastern Europe, Baltic states, and Israel). Within each region, we examined the percentage of centers which indicated that the particular treatment was their general policy. In addition, we assessed the influence of geographic region on treatment decision by performing logistic regression analysis with treatment as the dependent variable (general policy yes/no) and geographic region (categorical variable) as an independent variable. The Nagelkerke *R* ^2^ was reported, representing the proportion of variation in treatment that can be explained by geographic region. Analyses were performed using IBM SPSS Statistics version 21 software [17].

## Results

### Participating centers

Sixty-six centers (97% response rate) completed the questionnaire on ICP monitoring and treatment in patients with severe TBI. Questionnaires were completed mainly by intensive care physicians (*n* = 33, 50%) and neurosurgeons (*n* = 23, 35%). Most centers (*n* = 60, 91%) had an academic affiliation, and 44 (67%) were designated level I trauma centers (*see* Additional file 2 for definitions). Centers had a median of 33 (IQR 22–44) ICU beds in total and treated a median of 92 (IQR 52–160) patients with severe TBI annually. Forty-three (65%) centers had adopted a “closed” ICU model, which is defined as an ICU model where a critical care physician (intensivist) is primarily responsible for the delivery of care for patients at the ICU [18]. An “open” model, defined as an ICU model where the admitting physician (e.g., neurosurgeon) is primarily responsible for the care of ICU patients, was adopted in three (5%) centers [18]. A “mixed” model, which is an ICU model where the admitting physician is primarily responsible but the care is provided by a critical care physician, was adopted in 20 (30%) centers. Approximately half (*n* = 39) of the centers had a dedicated neurosciences ICU. Approximately three-fourths of sites (*n* = 49, 74%) indicated that they used the 2007 Brain Trauma Foundation (BTF) guidelines or institutional guidelines that were based on the BTF guidelines.

### Indications for ICP monitoring

**Fig. 1:**
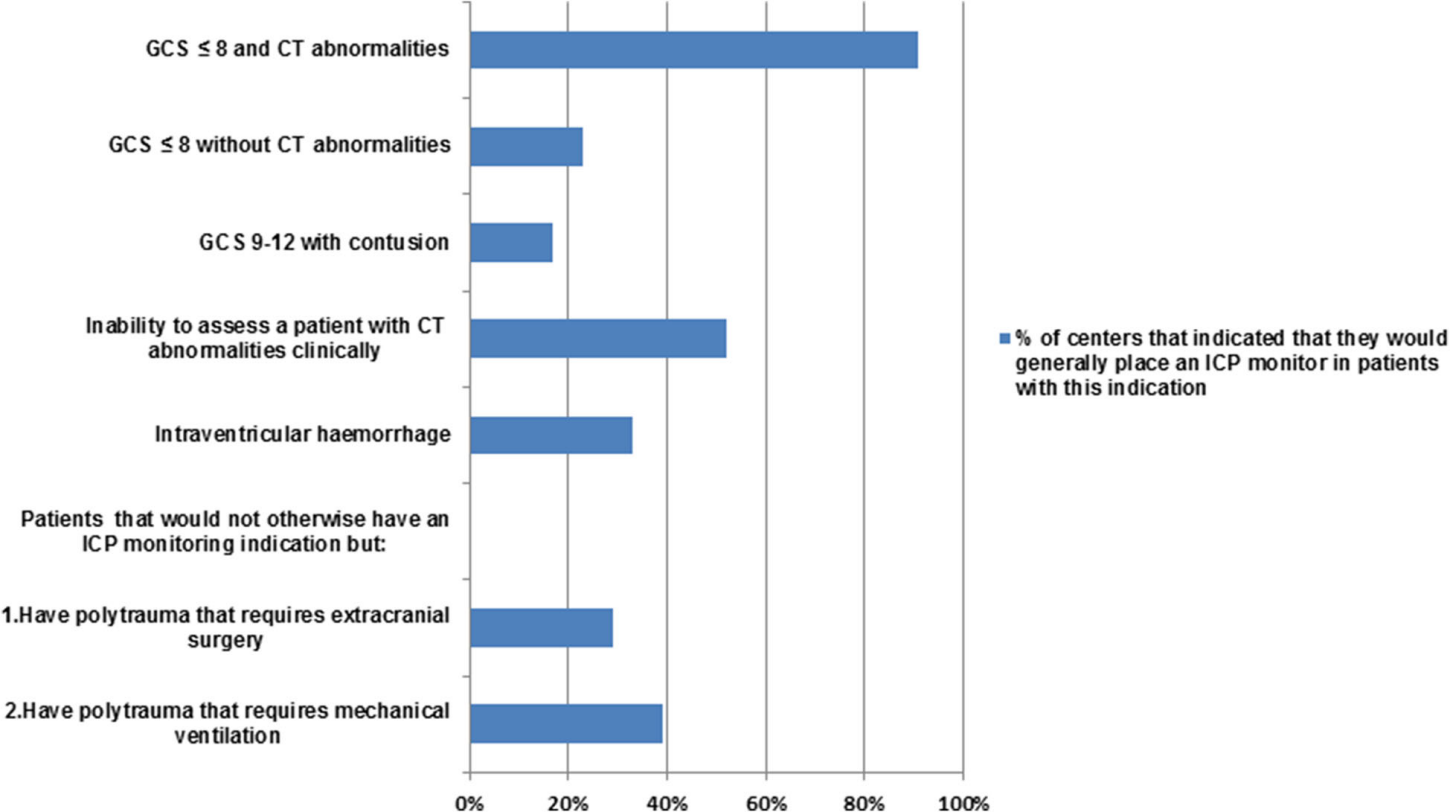
Indications for ICP monitoring placement. Shown are the percentages of centers that indicated that they would generally place an ICP monitor in patients with the described characteristics. Question was completed by 64 of 66 centers. *CT* Computed tomographic, *GCS* Glasgow Coma Scale, *ICP* Intracranial pressure

The majority of participants (*n* = 58, 91%) indicated that they would generally place an ICP monitor in patients with GCS ≤ 8 and CT abnormalities (Fig. 1). ICP monitors were less often considered for other indications, such as GCS ≤ 8 without CT abnormalities (*n* = 15, 23%), inability to assess a patient with CT abnormalities clinically (e.g., due to sedatives; *n* = 11, 17%), and intraventricular hemorrhage (*n* = 21, 33%). Around one-third of the participants would place an ICP monitor in patients with polytrauma (GCS > 8) who require extracranial surgery or mechanical ventilation but would not otherwise have an indication for ICP monitoring. Patient-specific reasons for not monitoring ICP included when the risk of raised ICP was considered low (*n* = 40, 62%), patients were considered unsalvageable (*n* = 37, 57%), or GCS was > 8 (*n* = 37, 57%) (Additional file 2).

### Variability in monitoring and treatment of intracranial hypertension

**Fig. 2:**
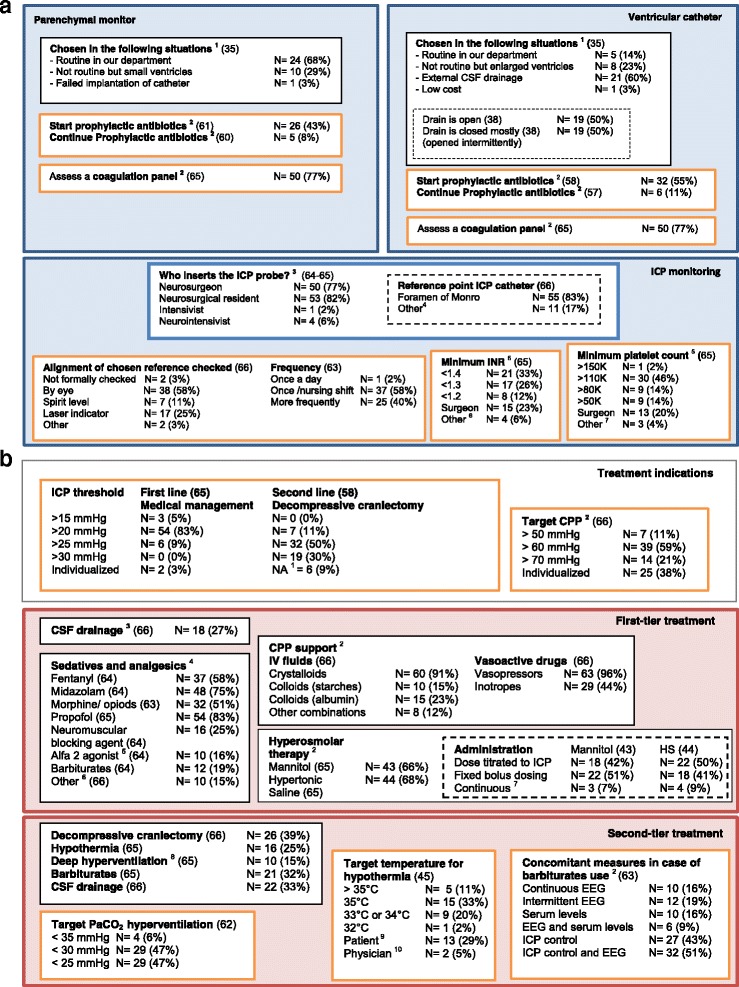
**a** Algorithm for ICP management: ICP monitoring. The *blue box* represents ICP monitoring with the policy of parenchymal monitor on the left and ventricular catheter on the right. *Orange boxes* are checkpoints during the ICP monitoring process. The *N* value represents the number of centers that indicated this answer as general policy with a corresponding percentage. The number in parentheses after the titles represents the number of centers that completed this question. ^1^ Centers that indicated these situations as the top one of the top three reasons for choosing a ventricular or parenchymal catheter. ^2^ Frequently and always summed. ^3^ Arterial blood pressure, midauricular level, ventricular motor, not applicable (we use only parenchymal monitors), room air, calibrated by device and meatus externa. ^4^ Prior to insertion of ventricular catheter for ICP monitoring. ^5^ Depending on other factors, such as the use of platelet aggregation inhibitors. ^6^ Multiplate and rotational thromboelastometric analysis prior to surgery if concerns. **b** Algorithm for ICP management: treatment indications, first- and second-tier treatment. The *red box* represents ICP treatment with first-tier treatment on *top* and second-tier treatment at the *bottom. Orange boxes* are checkpoints during the ICP treatment process. The *N* value represents the number of centers that indicated this answer as general policy with a corresponding percentage. The number in parentheses after the titles represents the number of centers that completed this question. ^1^ Decompressive craniectomy is (almost) never performed in our hospital. ^2^ Multiple answers were possible. ^3^ Only if ventricles are enlarged. ^4^ Frequently and always summed. ^5^ Clonidine or dexmedetomidine. ^6^ Sufentanil (4), remifentanil (2), β-blockers (1), alfentanil (2), esketamine (1). ^7^ Standard continuous infusion. ^8^ PaCO_2_ < 30 mmHg. ^9^ Variable, depends on patient. ^10^ Variable, depends on physician. *CPP* Cerebral perfusion pressure, *CSF* Cerebrospinal fluid, *EEG* Electroencephalogram, *HS* Hypertonic saline, *ICP* Intracranial pressure, *INR* International normalized ratio, *IV* Intravenous, *PaCO*_*2*_ Partial pressure of carbon dioxide

There is large variation in monitoring and treatment characteristics among European centers treating patients with TBI (Fig. 2a and b).

#### Parenchymal and ventricular ICP devices

Both parenchymal and ventricular ICP devices were available in more than half of centers (*n* = 38, 59%). One-third (*n* = 21) of the participants indicated that they used only parenchymal monitors, whereas five (8%) participants indicated that they used only ventricular catheters. In centers that used both types of monitors, parenchymal monitors were typically used routinely, with ventricular catheters placed either when the ventricles were enlarged or when cerebrospinal fluid (CSF) drainage was indicated. When a ventricular drain was used, half of the participants indicated that their local practice was generally to leave the drain open (*n* = 19, 50%), and the other half indicated a policy of intermittent drainage (*n* = 19, 50%) (Fig. 2a).

#### Precautions with ICP monitor placement

Half of the participants (55% ventricular catheter and 43% parenchymal sensor) indicated that they generally administered prophylactic antibiotics prior to the insertion of an ICP monitor, which was continued in around 10% of the centers. The majority of participants (*n* = 50, 77%) generally assessed the patient’s coagulation status prior to ICP monitor insertion. There was wide variability regarding the minimum international normalized ratio and minimum platelet count considered safe for device insertion (Fig. 2a).

#### Additional neuromonitoring

Half of the participants (*n* = 33) indicated that they generally used at least one additional neuromonitoring device (Additional file 2). Transcranial Doppler was generally applied in 24 (38%) centers, and brain tissue oxygenation was used in 12 (19%) centers.

#### First-tier treatment of elevated ICP

The majority of participants indicated an ICP threshold for medical treatment > 20 mmHg (*n* = 54, 83%) (Fig. 2b). There was less consensus on cerebral perfusion pressure (CPP) treatment thresholds; 39 participants (59%) indicated a threshold of 60 mmHg in their center, whereas 25 (38%) indicated individualized CPP targets.

Propofol (*n* = 54, 83%), midazolam (*n* = 48, 75%), fentanyl (*n* = 37, 58%), and morphine (*n* = 32, 51%) were generally used as part of first-tier treatment in patients with elevated ICP, whereas the use of α_2_-agonists (*n* = 10, 16%) and barbiturates (*n* = 12, 19%) was less frequent (Fig. 2b and Additional file 2). Neuromuscular blocking agents were generally used in 16 (25%) centers. Participants typically preferred a specific combination of sedatives and analgesics as part of first-tier treatments: 50 participants (76%) indicated they used two to four of eight sedatives and analgesics as general policy and the other interventions only infrequently (Additional file 2).

Regarding the use of osmotic therapy, two-thirds of the participants indicated generally using mannitol (*n* = 43, 65%) and/or hypertonic saline (*n* = 44, 67%). Seventeen participants indicated the use of mannitol, but not hypertonic saline, as their general policy, whereas 18 participants indicated the opposite. Fourteen (22%) participants indicated generally using hypertonic saline in conjunction with mannitol (Fig. 2b). Crystalloids were the most commonly used intravenous fluids to augment CPP (*n* = 60, 91%), whereas other fluids (starches, albumin, and other combinations) were less often used (12–23%). Vasopressors were generally used in almost all centers to support CPP (*n* = 63, 96%). Among the parameters used to titrate vasoactive drugs, mean arterial pressure targets (*n* = 51, 77%) and transpulmonary thermodilution monitoring by means of pulse contour cardiac output (*n* = 35, 53%) were most often used (Additional file 2).

#### Second-tier treatments for refractory intracranial hypertension

Among the second-tier treatments, decompressive craniectomy (*n* = 26, 39%), barbiturates (*n* = 21, 32%), and CSF drainage (*n* = 22, 33%) were the most often employed (Fig. 2b). Hypothermia and hyperventilation (partial pressure of carbon dioxide < 30 mmHg) were the general policy in 24.6% and 15.4% of the centers, respectively, whereas approximately one-third of the participants indicated never using hypothermia and hyperventilation (Additional file 2). Participants typically preferred one (*n* = 27, 42%) or two (*n* = 20, 31%) second-tier treatments and indicated use of the other options infrequently (Additional file 2). Details on indication, administration, and targets of second-tier treatments are presented in Additional file 2 and show a high degree of variability.

### Factors associated with aggressive monitoring and treatment policies

Around half of the centers were classified as using an aggressive ICP monitoring and treatment policy (*n* = 32, 48%). Centers with an open or mixed ICU model more often applied an aggressive ICP management style in comparison to centers with a closed ICU model (*p* = 0.05). We did not find significant associations between aggressiveness and any of the other factors studied (Table 1).

**Table 1:** Factors associated with an aggressive ICP management style

Factor	Relatively aggressive centers (*n* = 32)	Relatively conservative centers (*n* = 34)	*p* Value
ICU organization			0.05
Closed	17 (40%)	26 (60%)	
Open/mixed	15 (65%)	8 (35%)	
Dedicated neurosciences ICU			0.96
Available	19 (49%)	20 (51%)	
Not available	13 (48%)	14 (52%)	
BTF guidelines used^a^			0.48
Yes	25 (51%)	24 (49%)	
No	7 (41%)	10 (59%)	
Volume^b^			0.82
High volume	17 (47%)	19 (53%)	
Low volume	15 (50%)	15 (50%)	
Country’s income level^c^			0.83
High income	27 (49%)	28 (51%)	
Relatively low income	5 (46%)	6 (54%)	
Geographic location^d^			0.84
Northern Europe	4 (44%)	5 (56%)	
Western Europe	13 (52%)	15 (48%)	
United Kingdom	3 (43%)	4 (57%)	
Southern Europe	5 (42%)	7 (58%)	
Baltic states	2 (40%)	3 (60%)	
Eastern Europe	3 (50%)	3 (50%)	
Israel	2 (100%)	0 (0%)	

*BTF* Brain Trauma Foundation, *ICU* Intensive care unit

^a^ BTF guidelines or institutional guidelines that were broadly based on the BTF guidelines

^b^ Relatively high volume (number of patients with severe TBI admitted to the ICU higher than the median number of patients with severe TBI admitted to the ICU [*n* = 92]) vs. relatively low volume (number of patients with severe TBI admitted to the ICU lower than or equal to the median number of patients with severe TBI admitted to the ICU)

^c^ The division into relatively high- and low-income countries was based on a 2007 report by the European Union [21]. High income = Austria, Belgium, Denmark, Finland, France, Germany, Israel, Italy, The Netherlands, Norway, Spain, Sweden, United Kingdom, and Switzerland; relatively low income = Bosnia and Herzegovina, Bulgaria, Hungary, Latvia, Lithuania, Romania, and Serbia

^d^ Northern Europe = Norway, Sweden, Finland, and Denmark; Western Europe = Austria, Belgium, France, Germany, Switzerland, and The Netherlands; Southern Europe = Italy and Spain; Eastern Europe = Hungary, Romania, Serbia, and Bosnia and Herzegovina; Baltic states = Latvia and Lithuania

### Influence of geographic region on treatment decisions

The use of first- and second-tier treatments varied substantially within and between geographic regions (Table 2). Morphine and CSF drainage showed the largest within-region variation, with approximately half of the participants within each region stating they generally use these treatments. Between-region differences were especially pronounced for barbiturates as first-tier treatment. Barbiturates were used mainly in the Baltic states and Eastern Europe, and geographic region explained 63% of the variance in barbiturate use. In addition, the use of mannitol varied substantially across regions, with all participants in the Baltic states, Eastern Europe, and Israel indicating they generally use mannitol, whereas only 11% of the participants in Northern Europe stated they generally use mannitol. In Northern Europe, Western Europe, and the United Kingdom, propofol, midazolam, morphine, and hypertonic saline are generally applied as first-tier treatment, whereas participants in Southern Europe, the Baltic states, and Eastern Europe also indicated they generally use fentanyl, barbiturates, CSF drainage, and mannitol.

**Table 2:** Within- and between-region variation in first- and second-tier treatments for elevated intracranial pressure

Variable	Northern Europe (*n* =9)	Western Europe (*n* = 25)	United Kingdom (*n* =7)	Southern Europe (*N* =12)	Baltic states (*n* = 5)	Eastern Europe (*n* = 6)	Israel (*n* = 2)	Nagelkerke *R* ^2^ value
First-tier treatments								
Propofol	78%	76%	100%	92%	80%	67%	100%	0.14
Midazolam	67%	76%	29%	75%	100%	83%	100%	0.22
Fentanyl	44%	44%	29%	67%	100%	100%	50%	0.31
Morphine	56%	48%	57%	50%	40%	33%	50%	0.02
Neuromuscular blocking agents	0%	16%	29%	25%	40%	67%	50%	0.25
α_2_-Agonists	33%	12%	0%	17%	40%	0%	0%	0.22
Barbiturates	11%	8%	0%	0%	80%	83%	0%	0.63
CSF drainage	33%	24%	0%	25%	60%	50%	0%	0.20
Mannitol	11%	67%	43%	83%	100%	100%	100%	0.46
Hypertonic saline	89%	71%	86%	58%	40%	33%	100%	0.20
Second-tier treatments								
Decompressive craniectomy	33%	36%	29%	33%	80%	33%	100%	0.16
Hypothermia	22%	25%	71%	25%	0%	0%	0%	0.29
Deep hyperventilation	0%	13%	0%	33%	20%	33%	0%	0.24
Barbiturates	11%	29%	14%	33%	80%	67%	0%	0.25
CSF drainage	56%	28%	43%	33%	20%	17%	50%	0.08

*CSF* Cerebrospinal fluid

*Note.* Table presents the percentage of participants within each geographic region that indicated that the first- or second-tier treatment was their general policy. Nagelkerke *R* ^2^ was derived from a logistic regression analysis with treatment (general policy yes/no) as the dependent variable and geographic region (categorical variable) as an independent variable. Nagelkerke *R* ^2^ represents the proportion of variance of the treatment variable that is accounted for by geographic region

Northern Europe = Norway, Sweden, Finland, and Denmark; Western Europe = Austria, Belgium, France, Germany, Switzerland, and The Netherlands; Southern Europe = Italy and Spain; Eastern Europe = Hungary, Romania, Serbia, and Bosnia and Herzegovina; Baltic states = Latvia and Lithuania

## Discussion

We found substantial variation in the general approaches to ICP monitoring and treatment among 66 European neurotrauma centers. The majority of centers indicated that they would insert an ICP monitor in patients with severe TBI and head CT abnormalities. There was no consensus on other indications, however, nor was there consensus on peri-insertion precautions. The use of both first- and second-tier treatments for elevated ICP varied widely between centers and regions. We found that half of the centers employed a relatively aggressive ICP management approach, and the other half reported using a more conservative approach.

Strengths of this study include the high response rate (97%), the extensive development process of the questionnaire, and the comprehensive examination of both monitoring and treatment. In addition, because our survey was completed by centers that are currently collecting patient-level data for the CENTER-TBI study, the results of this study can be used directly as input for the CER analyses once the patient-level data become available. A limitation of our study is that the included centers represent a select group of European neurotrauma centers that are prominent in the field of neurotrauma care and research. Consequently, the picture obtained might be skewed. In addition, this study is dependent on perceived practices rather than on clinical data. Although we repeatedly emphasized confidentiality of results, we cannot exclude that some physicians presented (even subconsciously) a more favorable image or presented individual treatment preferences rather than the general policy in a center. This can be explored when individual patient-level data are available. A further limitation is that we asked for isolated general treatments but did not assess specific combinations. In clinical practice, however, different treatments are used simultaneously, and outcome might be determined by the combination of treatments provided rather than by one particular intervention.

The substantial variation in strategies for ICP management in our study was in line with previous survey studies in Europe [12, 13] and the United States [15]. For example, Hesdorffer et al. [15] found that mannitol, hypertonic saline, and hyperventilation were generally used in half of their centers. Guidelines have been proposed to reduce treatment variation in medicine [19]. Although there has been an increase in protocolization of medicine and awareness of guidelines during the last decade, variation in ICP management may not have been reduced [12, 13]. Moreover, some participants claimed using treatments that are discouraged in the BTF guidelines. For example, one-fifth of the participants specified using barbiturates as first-tier treatment, whereas this is a second-tier treatment in the BTF guidelines [20]. The discrepancy between BTF guidelines and reported policies indicates that there is little consensus among neurotrauma centers with respect to ICP management. This might be due to the relatively small evidence base underpinning the guidelines [3].

Our study has several implications for the planned CER analyses. We found wide variation for most of the topics studied, which enables analyzing effectiveness of ICP management at the hospital level. Analyzing effectiveness at the hospital level might be especially useful for treatments that were indicated to be used “rarely,” “sometimes,” or “frequently” by the large majority of participants. For these treatments, patient characteristics play an important role, and these can dramatically confound conventional patient-level analyses [4, 5]. Caution should be applied, however, in the interpretation of the effects of treatments that are performed solely in some regions and not in others. For example, barbiturates are often used as first-tier treatment in the Baltic states and Eastern Europe but not in other regions. A harmful or beneficial effect could therefore also be attributed to other aspects of care in the particular regions rather than to barbiturate use itself. In principle, it is possible to adjust statistically for between-center differences other than the treatment variable of interest with a random-effects model with a random intercept for center. However, when correlations between the treatment variable of interest and other factors that differ between centers are strong, such as for the first-line use of barbiturates and region, this might not be sufficiently captured by the random-effects model. In such a case, differences in outcome cannot be attributed with certainty to the treatment under study.

On the basis of our findings, we recommend prioritizing the following topics for CER because of the feasibility of the center-level approach:

1. ICP monitoring in patients with indications other than GCS ≤ 8 and CT abnormalities
2. Parenchymal vs. ventricular monitoring (with and without CSF drainage)
3. Use of first-tier treatments for elevated ICP (including use of neuromuscular blocking agents, mannitol vs. hypertonic saline vs. mannitol + hypertonic saline, fentanyl vs. no fentanyl, fluid management)
4. Use of second-tier treatments (including decompressive craniectomy vs. barbiturates vs. hypothermia)
5. The effect of an aggressive ICP management policy vs. a more conservative approach

## Conclusions

Substantial variation was found in the monitoring and treatment of patients with severe TBI and intracranial hypertension. These results indicate a lack of consensus among European neurotrauma centers and provide an important opportunity and necessity for CER to support the development of optimal treatment protocols for these severely affected patients.

## Additional files

Additional file 1: Provider profiling questionnaire. (PDF 672 kb)

Additional file 2: Detailed results. (PDF 85 kb)

## Abbreviations

BTF: Brain Trauma Foundation
CENTER-TBI: Collaborative European Neurotrauma Effectiveness Research in Traumatic Brain Injury
CER: Comparative effectiveness research
CPP: Cerebral perfusion pressure
CSF: Cerebrospinal fluid
CT: Computed tomographic
EEG: Electroencephalogram
GCS: Glasgow Coma Scale
HS: Hypertonic saline
ICP: Intracranial pressure
ICU: Intensive care unit
INR: International normalized ratio
IV: Intravenous
PaCO_2_: Partial pressure of carbon dioxide
RCT: Randomized controlled trial
TBI: Traumatic brain injury
