# Supplementary material for: Variation in monitoring and treatment policies for intracranial hypertension in traumatic brain injury: a survey in 66 neurotrauma centers participating in the CENTER-TBI study
Source: Crit Care. 2017 Sep 6;21:233. doi: 10.1186/s13054-017-1816-9 (PMC5586023; doi:10.1186/s13054-017-1816-9)
Supplement: Supplementary file 1 — Provider profiling questionnaire. (PDF 672 kb) [file 13054_2017_1816_MOESM1_ESM.pdf]

Additional file 1. Provider profiling questionnaire

GENERAL QUESTIONS

1.What type of hospital is your hospital?

- Academic / University hospital
- Nonacademic hospital

Academic / University Hospital = when your hospital is part of or affiliated to a University. An Academic/University hospital aims not only to deliver high-standard patient care, but also contributes to research and education

2.Is your hospital officially designated as trauma centre?

- No
- Yes
- N/A in our country/region

In some countries, hospitals designated as trauma centres are categorized, for example level 1 or 2 trauma centre. In other countries, these categories do not apply. If you live in a country that does not explicitly designate trauma centres, please select the N/A box.

3.If your hospital <u>is</u> a designated Trauma Centre, what is the designation level?

- Level I
- Level II
- Level III

4. How many Intensive Care Unit (ICU) beds does your hospital have in total (excluding beds that are exclusively the coronary care)?

……………………………………..

5. Does your hospital have a dedicated neuro-intensive care?

- No
- Yes

6.Which of the following best describes the model of ICU care at your hospital?
Please select one option only

- Closed ICU: critical care physicians (intensivists) assume primary responsibility for delivery of intensive care for Traumatic Brain Injury (TBI) patients. It is possible here that other specialists (e.g. a neurosurgeon) are consulted for advice when deterioration occurs. However, the intensivist remains responsible
- Open ICU: the admitting surgeon (neurosurgeon / trauma surgeon) assumes primary responsibility for care of TBI patients, including the provision of critical care services. This model of care may include elective consultation of an intensivist
- Mixed: the admitting surgeon (neurosurgeon / trauma surgeon) assumes primary responsibility for care of TBI patients. A certified physician in critical care (intensivists) coordinates the delivery of care

7.What is the number of Traumatic Brain Injury (TBI) patients treated in your Intensive Care Unit (ICU) annually?

1. 2012: ……………………………………..
2. 2013: ……………………………………..

8. With reference to guidelines for Intensive Care Unit (ICU) management of Traumatic Brain Injury (TBI), does your ICU:

- Not have specific guidelines for management
- Follow the Brain Trauma Foundation Guidelines
- Follow National Guidelines (Please specify: ……………………………………………)
- Have institutional guidelines which are broadly based on BTF and/or National Guidelines
- Have separate guidelines which you have developed independently

(if #4 or #5 above, please upload a copy of your guidelines)

QUESTIONS ABOUT ICP MANAGEMENT

ICP monitoring

1. What are indications for ICP monitoring in your hospital?
Please provide us the general clinical practice at your center. This does not have to be the same as stated in the guidelines you use

This question is about indications for ICP monitoring in your center.

Select CONSIDERED NOT IMPORTANT IN DECISION MAKING in factors that are never a reason for ICP monitoring.

Select ONLY IN THE PRESENCE OF OTHER RISK FACTORS if the factor is never solely a reason for ICP monitoring, but it might be a reason in combination with one or more other risk factors. For example: a hospital may perform ICP monitoring in patients with a GCS ≤ 8 without CT abnormalities if there are other risk factors present. Respondents from such a hospital should tick 'only in the presence of other risk factors' after GCS ≤ 8.

Select GENERAL POLICY when the criteria are, in general, a reason for ICP monitoring in your hospital. When you select GENERAL POLICY this must represent a general consensus among colleagues, rather than individual preference.

Where you are in doubt whether this is the appropriate response to the question, we would recommend, for example, either a verbal discussion or an email exchange with colleagues to check consensus.

	Considered not important in decision making	Only in the presence of other risk factors	General Policy
GCS ≤ 8 and CT abnormalities	☐	☐	☐
GCS ≤ 8 without CT abnormalities	☐	☐	☐
GCS 9-12 with contusion	☐	☐	☐
Inability to assess a patient with CT	☐	☐	☐

abnormalities clinically (e.g. sedation, surgery etc.)			
Intraventricular haemorrhage	☐	☐	☐
Other, please specify………………………	☐	☐	☐

2. What are reasons for NOT monitoring ICP at your Intensive Care Unit (ICU)?

	Never (0-10%)	Rarely (10-30%)	Sometimes (30-70%)	Frequently (70-90%)	Always (90-100%)
Glasgow Coma Scale (GCS) > 8	☐	☐	☐	☐	☐
No radiological signs of raised ICP	☐	☐	☐	☐	☐
Risk of raised ICP considered low	☐	☐	☐	☐	☐
Patient considered unsalvageable	☐	☐	☐	☐	☐
Coagulopathy (non-drug related)	☐	☐	☐	☐	☐
Use of anticoagulants or platelet aggregation inhibitors	☐	☐	☐	☐	☐
No device available	☐	☐	☐	☐	☐
Not local policy to monitor ICP	☐	☐	☐	☐	☐
We adhere to a protocol in which treatment is based on imaging and clinical examination	☐	☐	☐	☐	☐
Too costly	☐	☐	☐	☐	☐
Other, please specify……	☐	☐	☐	☐	☐

The responses to this question should represent, as best as practicable, a general consensus on treatment at your center, rather than individual management preferences or opinions.

3. Is there structural variation between (neuro)surgeons within your hospital with regard to the decision to place an ICP sensor?

- No
- Yes

Structural variation refers to a situation in which one or more of the neurosurgeons are generally more likely to place an ICP sensor than others.

4. When a patient with polytrauma and minor intracranial pathology (which would not otherwise indicate ICP monitoring) requires <u>extracranial surgery</u> which is not life-saving, in the acute phase after trauma, do you:
Select all that apply

- ☐ Place an ICP monitor and allow surgery to proceed
- ☐ Repeat a CT scan before/after surgery
- ☐ Undertake a sedation hold before and/or after surgery
- ☐ Postpone surgery if at all possible
- ☐ Other, please specify…..

The responses to this question should represent, as best as practicable, a general consensus on treatment at your center, rather than individual management preferences.

5. In polytrauma patients with a Glasgow Coma Scale (GCS) >8 and small but not severe initial CT abnormalities, who require <u>mechanical ventilation</u> for a number of days because of extracranial injuries, we apply ICP monitoring:

- o Never
- o Only sometimes
- o Often
- o Always

The responses to this question should represent, as best as practicable, a general consensus on treatment at your center, rather than individual management preferences.

6. What kind of ICP sensors are used in your hospital?

- o Parenchymal monitors without optional ventricular drainage
- o Ventricular catheters
- o Both
- o Not applicable since ICP sensors are not used

6b. In case you answered 'both' in the previous question:
If you use parenchymal and ventricular catheters in your hospital, when would you use ventricular/ventricular+ sensor monitors (instead of parenchymal monitoring)?

please rank the top 3 reasons:
1: …………..
2: ………….
3: …………..

- ☐ Routine in our department
- ☐ Not routine, but enlarged ventricles
- ☐ External CSF drainage
- ☐ No parenchymal device available
- ☐ Low cost
- ☐ Other, please specify…………………………………

6c. If you use parenchymal and ventricular catheters in your hospital, when would you use parenchymal monitors (instead of ventricular/ventricular+sensor)?

1: …………..
2: ………….
3: …………..

- ☐ Routine in our department
- ☐ Not routine, but small ventricles
- ☐ Mainly motivated by time of day
- ☐ No OR available for placement ventricular catheter
- ☐ Failed implantation ventricular cathether
- ☐ Other, please specify……………………………..

6d. When deciding to monitor ICP we routinely use additional ventricular CSF drainage:

- o No, never or seldom
- o As second tier therapy to control ICP
- o Only if ventricles are enlarged
- o Yes, always initially

6e. If you use a ventricular drain:

During the use of the ventricular drain, is the drain open or closed during the most of the time?

- ☐ Ventricular drain is open to drain CSF
  - o If this answer option is selected: At what level? ……………………………………………
- ☐ Ventricular drain is closed mostly and opened intermittently
  - o If this answer option is selected: At what pressure is this opened? And for how long?
- ☐ Other, please specify……………………………..

Intensive Care Unit (ICU) practice around ICP monitoring

	Never (0-10%)	Rarely (10-30%)	Sometimes (30-70%)	Frequently (70-90%)	Always (90-100%)	N/A, we do not have this technique
7. Are prophylactic antibiotics given prior to ICP monitor insertion?	Ventricular catheter:					
	○	○	○	○	○	○
	Parenchymal sensor:					
	○	○	○	○	○	○
8. Are prophylactic antibiotics continued after ICP monitoring insertion?	Ventricular catheter:					
	○	○	○	○	○	○
	Parenchymal sensor					
	○	○	○	○	○	○
9. Is a	Ventricular catheter:					

coagulation panel assessed prior to insertion of an ICP monitoring device?	○	○	○	○	○	○
	Parenchymal sensor					
	○	○	○	○	○	○

10. What is considered a minimum platelet count for insertion of a ventricular catheter in your Intensive Care Unit (ICU)?

- ○ >150K
- ○ >100K
- ○ > 80 K
- ○ >50K
- ○ Variable, depends on surgeon
- ○ No minimum
- ○ Other, please specify ………………………………….

11. What is consider the minimum INR for safe placement of a ventricular catheter in your Intensive Care Unit (ICU)?

- ○ <1.4
- ○ <1.3
- ○ <1.2
- ○ Variable, depending on surgeon
- ○ No minimum
- ○ Other, please specify…………………………………………………………..

12. Who inserts the catheter/probes for ICP monitoring?
Please provide us the general clinical practice at your center. This does not have to be the same as stated in the guidelines you use

This question is about indications for ICP monitoring in your center.

Select NEVER if the specialism has never inserted ICP monitors in TBI patients.

Select RARELY / EXCEPTIONAL if the specialism CAN insert ICP monitors and does this during exceptional circumstances (e.g. during the night, crisis, overcrowding).

Select GENERAL POLICY when the specialism usually inserts ICP monitors.

Where you are in doubt whether this is the appropriate response to the question, we would recommend, for example, either a verbal discussion or an email exchange with colleagues to check consensus.

	Never	Rarely / Exceptional	General Policy
Neurosurgeon	☐	☐	☐
Neurosurgical resident	☐	☐	☐
Intensivist	☐	☐	☐
Intensivist resident	☐	☐	☐
Neurointensivist	☐	☐	☐

Non-neurosurgical surgeon	☐	☐	☐
Physician assistant / nurse practitioner	☐	☐	☐
Other, please specify….	☐	☐	☐

13. At what level does your Intensive Care Unit (ICU) zero the ICP catheter?
    - o Foramen of Monro
    - o Same level as arterial blood pressure
    - o Other, please specify…………………………….

14. At what level does your Intensive Care Unit (ICU) zero the transducer for arterial blood pressure (for calculation of CPP)?
    - o Right atrium
    - o Level of arterial catheter
    - o Foramen of Monroe
    - o Other, please specify………………..

15. How is the alignment of the transducer to the chosen reference level checked?
    - o Not formally checked
    - o By eye
    - o Spirit level
    - o Laser indication
    - o Other (please specify)………………..

15b. If the level is formally assessed, is this done:
- o At admission
- o Once each day
- o Once per nursing shift
- o More frequently

CPP monitoring

16. Please list the target Cerebral Perfusion Pressure utilized at your facility:
Select all that apply
    - ☐ > 50 mmHg
    - ☐ > 60 mmHg
    - ☐ > 70 mmHg
    - ☐ Individualized

17. For treating CPP, which types of IV fluids are used to augment intravascular volume?
Select all that apply
    - ☐ Crystalloids
    - ☐ Colloids – starches
    - ☐ Colloids - albumin
    - ☐ Other combinations

18. Which vasoactive drugs are used to support CPP in patients with Traumatic Brain Injury (TBI)?
Select all that apply

- ☐ Vasopressors
- ☐ Inotropes
- ☐ Other, please specify………

19. What monitoring devices are used to titrate vasoactive drugs?
Select all that apply

- ☐ MAP targets only
- ☐ Central venous pressure
- ☐ PICCO
- ☐ Lidco
- ☐ Oesophageal Doppler monitor
- ☐ Pulmonary artery catheter
- ☐ Others, please specify ……

Advanced monitoring

20. Which of the following additional techniques are utilized at your Intensive Care Unit (ICU) for neuromonitoring?
Select all that apply

	Never (0-10%)	Rarely (10-30%)	Sometimes (30-70%)	Frequently (70-90%)	Always (90-100%)
Cerebral microdialysis	☐	☐	☐	☐	☐
Transcranial Doppler	☐	☐	☐	☐	☐
CBF probes	☐	☐	☐	☐	☐
Jugular Venous saturation monitors	☐	☐	☐	☐	☐
Near infrared monitors	☐	☐	☐	☐	☐
Brain tissue oxygenation	☐	☐	☐	☐	☐
Other, please specify………….	☐	☐	☐	☐	☐

Treatment of severe Traumatic Brain Injury (TBI) patients at the Intensive Care Unit (ICU)

21. At your Intensive Care Unit (ICU), the threshold for medical management of elevated ICP is:

- ○ > 15 mmHg
- ○ >20 mmHg
- ○ >25 mmHg
- ○ Other, please specify………………

22. At your Intensive Care Unit (ICU), the threshold for performing a decompressive craniectomy in patients with diffuse injury is:

- > 15 mmHg
- >20 mmHg
- >25 mmHg
- > 30 mm Hg
- NA. Decompressive craniectomy is (almost) never performed in our hospital

Sedation

23. Please rate the utilization of the following sedatives, neuromuscular blockers, and analgesics as first line therapies for ICU management of Traumatic Brain Injury (TBI) patients with increased ICP:

	Never (0-10%)	Rarely (10-30%)	Sometimes (30-70%)	Frequently (70-90%)	Always (90-100%)
Fentanyl	☐	☐	☐	☐	☐
Midazolam	☐	☐	☐	☐	☐
Morphine (or other opioids)	☐	☐	☐	☐	☐
Propofol	☐	☐	☐	☐	☐
Neuromuscular blocking agents	☐	☐	☐	☐	☐
Alfa 2 agonist (clonidine or dexmedetomidine)	☐	☐	☐	☐	☐
Barbiturates	☐	☐	☐	☐	☐
Other, please specify…..	☐	☐	☐	☐	☐

Hyperosmolar therapy (mannitol and/or hypertonic saline

Please provide us the general clinical practice at your center. This does not have to be the same as stated in the guidelines you use

This question is about the use of hyperosmotic therapy in patients with elevated ICP.

Select NEVER in agents that are never used in your center.

Select RARELY / EXCEPTIONAL in agents that can be used but are no general policy.

Select GENERAL POLICY when the agent is, in general, used to treat elevated ICP in your center.
When you select GENERAL POLICY this must represent a general consensus among colleagues, rather than individual preference.
Where you are in doubt whether this is the appropriate response to the question, we would recommend, for example, either a verbal discussion or an email exchange with colleagues to check consensus.

	Never	Rarely / Exceptional	General Policy
24. Is mannitol utilized to treat patients with increased ICP in your ICU ?	☐	☐	☐
25. Is hypertonic saline	☐	☐	☐

utilized to treat patients with increased ICP in your ICU?

26. Is hypertonic saline administered in conjunction with mannitol? ☐ ☐ ☐

Mannitol and Hypertonic Saline

	**Mannitol**	**Hypertonic saline**
27. How are these agents administered?	☐ N/A<br>☐ Dose titrated to ICP<br>☐ Fixed Bolus dosing<br>☐ Standard continuous infusion	☐ N/A<br>☐ Dose titrated to ICP<br>☐ Fixed Bolus dosing<br>☐ Standard continuous infusion
28. If administered in fixed bolus doses, how frequently is it given:	☐ N/A<br>☐ More often than 6 times per day<br>☐ 6 times per day (every 4 hours)<br>☐ 4 times per day (every 6 hours)<br>☐ Less than 4 times per day<br>☐ Other, please specify……………..	☐ N/A<br>☐ More often than 6 times per day<br>☐ 6 times per day (every 4 hours)<br>☐ 4 times per day (every 6 hours)<br>☐ Less than 4 times per day<br>☐ Other, please specify…………………….
	If mannitol is administered, is serum osmolarity monitored?<br>☐ N/A<br>☐ No<br>☐ Yes<br>If yes, an upper limit of ……….. mOsm/liter	If hypertonic saline is administered as a continuous infusion, is there a serum sodium goal?<br>☐ N/A<br>☐ No<br>☐ Yes<br>If yes, a goal of …………………(mEq/L)

The responses to this question should represent, as best as practicable, a general consensus on treatment at your center, rather than individual management preferences.

Second/third tier therapies for treatment of raised intracranial pressure

> The responses to the following questions should represent, as best as practicable, a general consensus on treatment at your center, rather than individual management preferences.

29a. Are the following approaches used to treat <u>refractory</u> intracranial hypertension?

	Never (0-10%)	Rarely (10-30%)	Sometimes (30-70%)	Frequently (70-90%)	Always (90-100%)
Decompressive craniectomy	☐	☐	☐	☐	☐
Hypothermia (temperature < 36 degrees Celsius)	☐	☐	☐	☐	☐
Intensive hyperventilation (PCO2 < 4.0 kPa)	☐	☐	☐	☐	☐
Barbiturates	☐	☐	☐	☐	☐

29b. If hyperventilation is answered with rarely – always:
When is hyperventilation (PaCO2 < 4,0 kpa)) utilized in Traumatic Brain Injury (TBI) patients at the intensive care unit?

	Never (0-10%)	Rarely (10-30%)	Sometimes (30-70%)	Frequently (70-90%)	Always (90-100%)
prophylactic hyperventilation (PaCO2 < 35 mmHg)	☐	☐	☐	☐	☐
To manage intracranial pressure for less than six hours	☐	☐	☐	☐	☐
To manage intracranial pressure for more than six hours	☐	☐	☐	☐	☐
In cases of imminent herniation	☐	☐	☐	☐	☐
Is brain tissue oxygen monitoring (PbtO2) used to measure cerebral oxygenation during hyperventilation?	☐	☐	☐	☐	☐

29c. If hyperventilation is used, what is the target PaCO2 (as second/third tier therapy)?

- <35 mmHg
- <30 mmHg
- <25 mmHg
- Variable, dependent on patient
- Variable, dependent on surgeon/intensivist

29d. If hypothermia is used, what is the target temperature?

- >35°
- 35°
- 33 or 34°
- 32°
- Variable depending on patient
- Variable depending on physician

29e. If barbiturates are used, how is the dose targeted?
Select all that apply

- ☐ Continuous EEG monitoring
- ☐ Intermittent EEG recording
- ☐ Serum levels
- ☐ EEG and serum levels
- ☐ ICP control
- ☐ ICP control and EEG
