# Supplementary material for: Variation in monitoring and treatment policies for intracranial hypertension in traumatic brain injury: a survey in 66 neurotrauma centers participating in the CENTER-TBI study
Source: Crit Care. 2017 Sep 6;21:233. doi: 10.1186/s13054-017-1816-9 (PMC5586023; doi:10.1186/s13054-017-1816-9)
Supplement: Supplementary file 2 — Detailed results. (PDF 85 kb) [file 13054_2017_1816_MOESM2_ESM.pdf]

Additional file 2. Detailed results

1.Definitions used in this paper

Academic hospital	A hospital that is part of or affiliated to a university. An academic hospital aims not only to deliver high-standard patient care, but also contributes to research and education.
First-tier treatments of elevated ICP	Intracranial pressure lowering therapies that are used when ICP is raised. These therapies include sedation, paralysis, cerebrospinal fluid removal, and osmotic therapy.
General Policy	The way the large majority of patients (>75%) with a particular indication would be treated. We are interested in a general consensus on treatment here, rather than individual treatment preferences.
ICU model	*Closed* – An ICU model where critical care physicians (intensivists) assume primary responsibility for delivery of intensive care for TBI patients. It is possible that other specialists (e.g. neurosurgeon) are consulted for advice when deterioration occurs. However, the intensivist remains responsible.<br>*Open* – An ICU model where the admitting surgeon (neurosurgeon / trauma surgeon) assumes primary responsibility for care of TBI patients, including the provision of critical care services. This model of care may include elective consultation of an intensivist<br>*Mixed* – An ICU model where the admitting surgeon (neurosurgeon / trauma surgeon) assumes primary responsibility for care of TBI patients. A certified physician in critical care (intensivists) coordinate the delivery of care
Level I trauma center	A regional resource center that generally serves large cities or population-dense areas. A level I trauma center is expected to manage large numbers of severely injured patients (at least 1,200 trauma patients yearly or have 240 admissions with an injury severity score of more than 14). It is characterized by 24-hour in-house availability of an attending surgeon and the prompt availability of other specialties (e.g. neurosurgeon, trauma surgeon).
Second-tier treatments or rescue therapies of elevated ICP	Approaches used to treat refractory intracranial hypertension (intracranial hypertension that does not respond to first-tier therapies). These include intensive hyperventilation, barbiturates, hypothermia and (surgical) decompressive craniectomy.
Structural variation	A situation in which a different policy exist within one center with regard to diagnostic and therapeutic decisions. For example one or more of the clinicians are generally more likely to place an ICP sensor.

Abbreviations. ICP = intracranial pressure; ICU = intensive care unit; TBI = traumatic brain injury.

2. Patient-specific reasons for not monitoring ICP

Contra-indication for ICP monitoring	N completed	Number of centers (%)*
GCS > 8	65	37 (57%)
No radiological signs of raised ICP	65	35 (54%)
Risk of raised ICP considered low	65	40 (62%)
Patient considered unsalvageable	65	37 (57%)
Coagulopathy (non-drug related)	65	16 (25%)
Use of anticoagulants or platelet aggregation inhibitors	65	16 (25%)
No local policy or center adheres to a protocol in which treatment is based on imaging and clinical examination	64	9 (14%)
Other contra indication‡	65	2 (3%)

‡Other contra indication: too costly or no device available

*Number of centers (%) that indicated that the criteria was frequently or always a contra-indication for the placement of an intracranial pressure (ICP) monitor.

Abbreviations. GCS = Glasgow Coma Scale; ICP = intracranial pressure.

3. Additional neuromonitoring

Technique	N completed	Number of centers (%)*
Cerebral microdialysis	65	4 (6%)
Transcranial Doppler	64	24 (38%)
Cerebral blood flow probes	63	3 (5%)
Jugular venous saturation monitors	64	6 (9%)
Near infrared monitors	64	1 (2%)
Brain tissue oxygenation	64	12 (19%)
OtherȽ	64	3 (5%)

*Number of centers that indicated that they frequently or always use this technique in patients with severe traumatic brain injury

ȽOther: EEG, non invasive PRx monitor, ICM+

4. Frequency of first-tier therapies that are used to treat severe TBI patients with elevated ICP

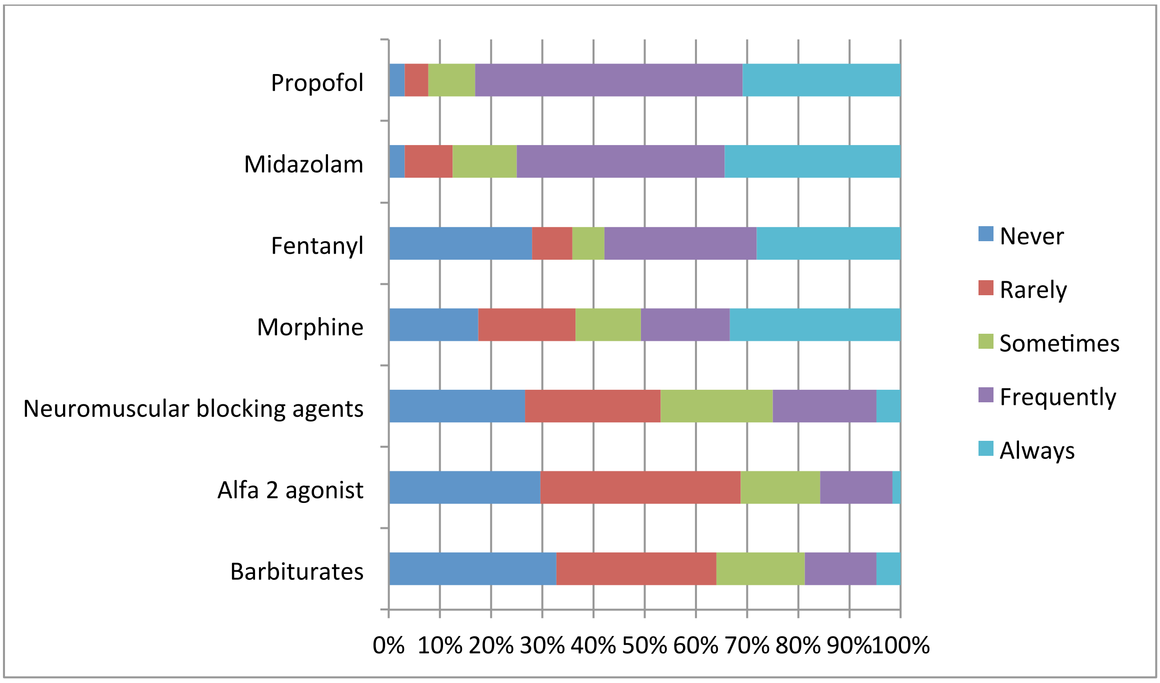

5. Number of first-tier treatments that are generally (frequently or always) used for the treatment of elevated intracranial pressure

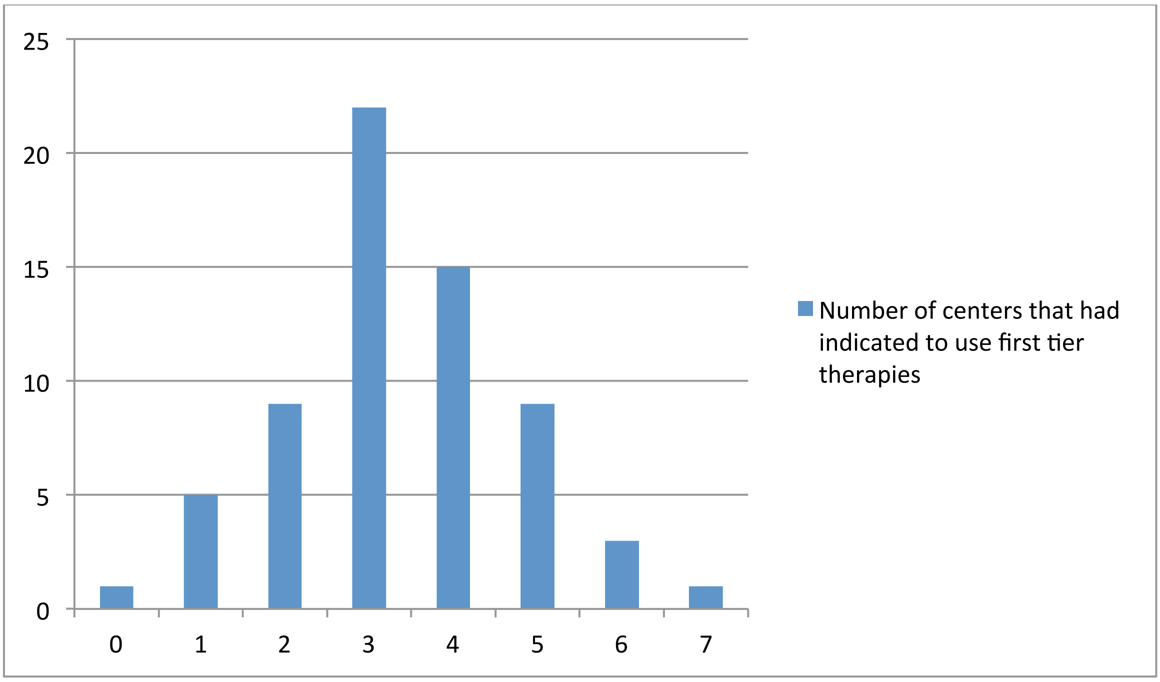

Figure presents the number of first-tier therapies that are generally used by the participating centers. On the x-axis the number of first-tier therapies (range 1-7) are presented and on the y-axis the number of centers (total: 66) are presented.

6. Parameters that are used to titrate vasoactive drugs

Device	N completed	Number of centers (%)*
Mean arterial pressure targets	66	51 (77%)
Central venous pressure	66	31 (47%)
Pulse index contour continuous cardiac output (PICCO)	66	35 (53%)
LIDCO	66	3 (5%)
Oesophageal Doppler monitor	66	5 (8%)
Pulmonary artery catheter	66	12 (18%)
OtherȽ	66	10 (15%)

*Number of center that indicated that they used the device to titrate vasoactive drugs; More than one answer was possible, as long as it represents the generally policy

ȽOther: central venous oxygen saturation, echocardiography, ECO heart, sign of volemia (diuresis, capillary refill, pulsus paradoxus, lactate SvO2), transthoracic echo, TTE, ultrasound, vilgileo)

7. Frequency of the second-tier therapies (decompressive craniectomy, hypothermia, intensive hyperventilation and barbiturates) in the treatment of severe TBI patients with elevated ICP

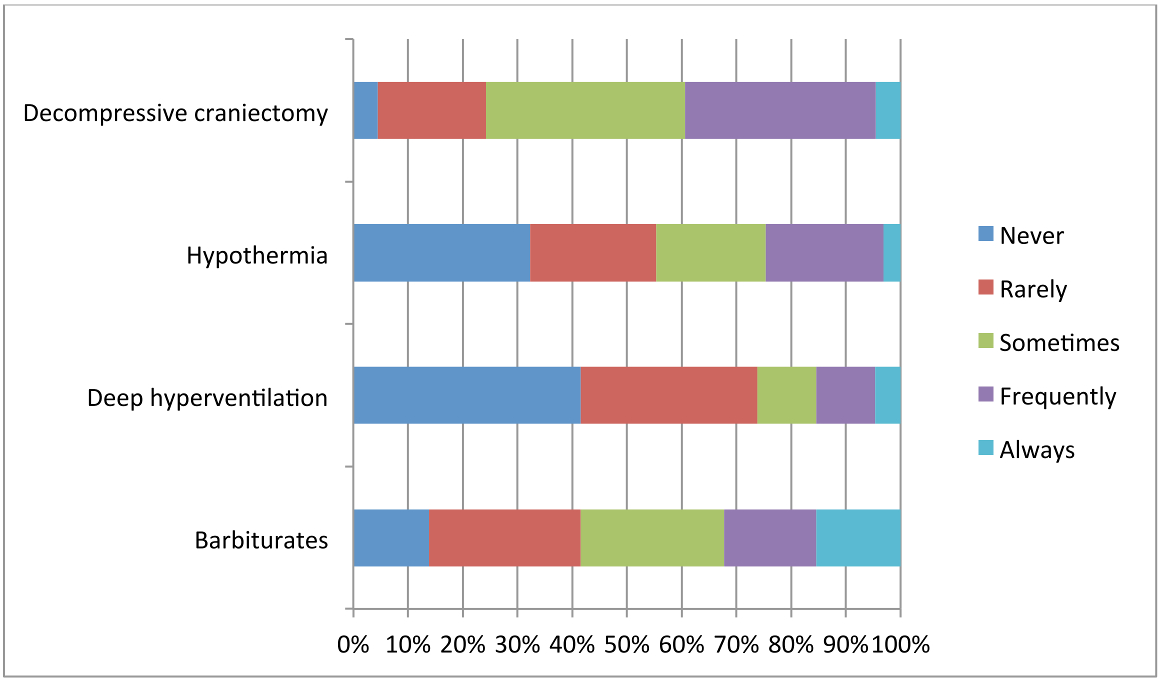

8. Number of second-tier treatments that are generally (frequently or always) used in combination for the treatment of elevated intracranial pressure

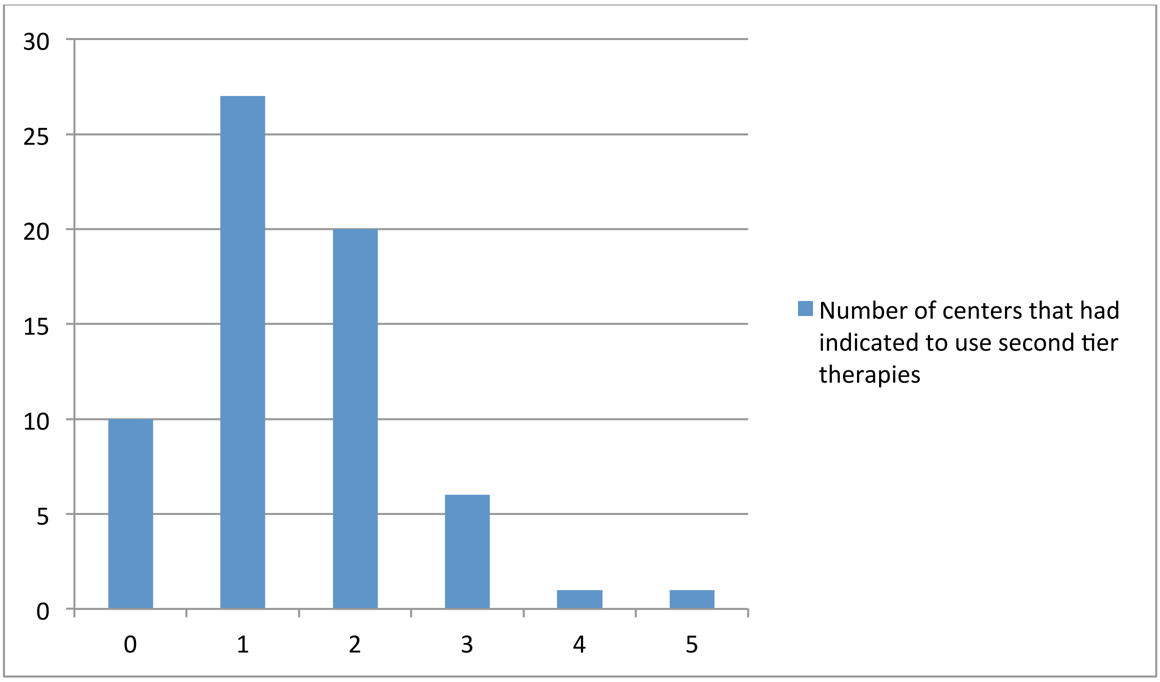

Figure presents the number of second-tier therapies that are generally used by the participating centers. On the x-axis the number of second-tier therapies (range 1-5) are presented and on the y-axis the number of centers (total: 66) are presented

9. Hyperventilation as second-tier treatment for elevated ICP

Characteristic	N completed	Number of centers (%)*
Indications for hyperventilation		
Prophylactic hyperventilation	39	5 (13%)
To manage intracranial pressure for less than 6 hours	38	6 (16%)
To manage intracranial pressure for more than six hours	39	3 (8%)
In case of imminent herniation	38	23 (61%)
Measurements during hyperventilation		
Brain tissue oxygen monitoring is used to measure cerebral oxygenation	38	8 (21%)

*Number of centers that indicated that they use apply the indication or the PaCO2 target frequently or always as second-tier therapies in patients with severe TBI
